# Genetic Modification of Brain Organoids

**DOI:** 10.3389/fncel.2019.00558

**Published:** 2019-12-17

**Authors:** Jan Fischer, Michael Heide, Wieland B. Huttner

**Affiliations:** Max Planck Institute of Molecular Cell Biology and Genetics, Dresden, Germany

**Keywords:** brain organoids, genetic modification, adeno-associated virus, electroporation, lentivirus, transposon, CRISPR/Cas9

## Abstract

Brain organoids have become increasingly used systems allowing 3D-modeling of human brain development, evolution, and disease. To be able to make full use of these modeling systems, researchers have developed a growing toolkit of genetic modification techniques. These techniques can be applied to mature brain organoids or to the preceding embryoid bodies (EBs) and founding cells. This review will describe techniques used for transient and stable genetic modification of brain organoids and discuss their current use and respective advantages and disadvantages. Transient approaches include adeno-associated virus (AAV) and electroporation-based techniques, whereas stable genetic modification approaches make use of lentivirus (including viral stamping), transposon and CRISPR/Cas9 systems. Finally, an outlook as to likely future developments and applications regarding genetic modifications of brain organoids will be presented.

## Introduction

The development of brain organoids (Kadoshima et al., 2013; Lancaster et al., 2013) has opened up new ways to study brain development and evolution as well as neurodevelopmental disorders. Brain organoids are multicellular 3D structures that mimic certain aspects of the cytoarchitecture and cell-type composition of certain brain regions over a particular developmental time window (Heide et al., 2018). These structures are generated by differentiation of induced pluripotent stem cells (iPSCs) or embryonic stem cells (ESCs) into embryoid bodies followed by, or combined, with neural induction (Kadoshima et al., 2013; Lancaster et al., 2013). In principle, two different classes of brain organoid protocols can be distinguished, namely: (i) the self-patterning protocols which produce whole-brain organoids; and (ii) the pre-patterning protocols which produce brain region-specific organoids (Heide et al., 2018). However, brain organoids are far from being ideal models of the brain, and notably cortical development (for a review, see Heide et al., 2018). The main issues concern reproducibility and the modeling of later stages of brain development, as mainly early stages of brain development, are modeled correctly. Very recently, approaches have been undertaken to improve the reproducibility through the optimization of protocols (Velasco et al., 2019). Moreover, modeling of later stages has been addressed by introducing organoid slice cultures grown at the air-liquid interface. This has resulted in increased neuronal survival and improved morphology as well as the generation of axonal tracts (Giandomenico et al., 2019). In future, brain organoid protocols are likely to result in even better 3D models of neural development, evolution and disease. An important step in this direction has been the very recent development of human brain organoids with a vascular-like system (Cakir et al., 2019).

However, even the best 3D model of brain development loses much of its usefulness if one cannot modify it. The ability to genetically modify brain organoids is essential for their utility as models of brain development (Birey et al., 2017), evolution (Mora-Bermúdez et al., 2016) and disease (Bian et al., 2018) as well as their evolving use as drug-screening platforms (Zhou et al., 2017). Genetic modification is a powerful tool that allows for the introduction of alterations ranging from small changes (e.g., point mutations) to the removal or integration of entire genes. This enables researchers to investigate individual genes as well as entire gene cassettes to elicit gene expression patterns, functions, and interactions. Moreover, drugs can be tested in a larger variety of disease states and in different genetic environments.

Rather than focusing on the discussion of the advantages, disadvantages and potential applications of the different brain organoid protocols, which have already been addressed in several excellent reviews (e.g., Kelava and Lancaster, 2016; Quadrato et al., 2016; Di Lullo and Kriegstein, 2017; Heide et al., 2018), the present review will focus on the genetic modification of brain organoids. An overview of the different types and methods of genetic modification in brain organoids will be given, and their advantages and disadvantages, as well as examples of their application, will be discussed.

## Types of Genetic Modification of Brain Organoids

Any method of genetic modification needs to address three key issues: (i) the nature of the genetic modification; (ii) the stage, within brain organoid development, at the time of genetic modification; and (iii) the target cells of the genetic modification.

The first issue, namely the nature of the genetic modification, concerns stable vs. transient genetic modification. In a stable modification, the genetic alteration is introduced into the genome of a cell and is thus passed on to future cell generations. In contrast, in transient genetic modifications, genetic cargo (e.g., genes, short interfering RNAs, etc.) is administered to a cell without genomic insertion and the possibility of further replication. The delivered genetic cargo is then progressively degraded and diluted with each cell division. For proliferating cells, in particular, this means that the level of the administered genetic material continuously declines.

**Figure 1:**
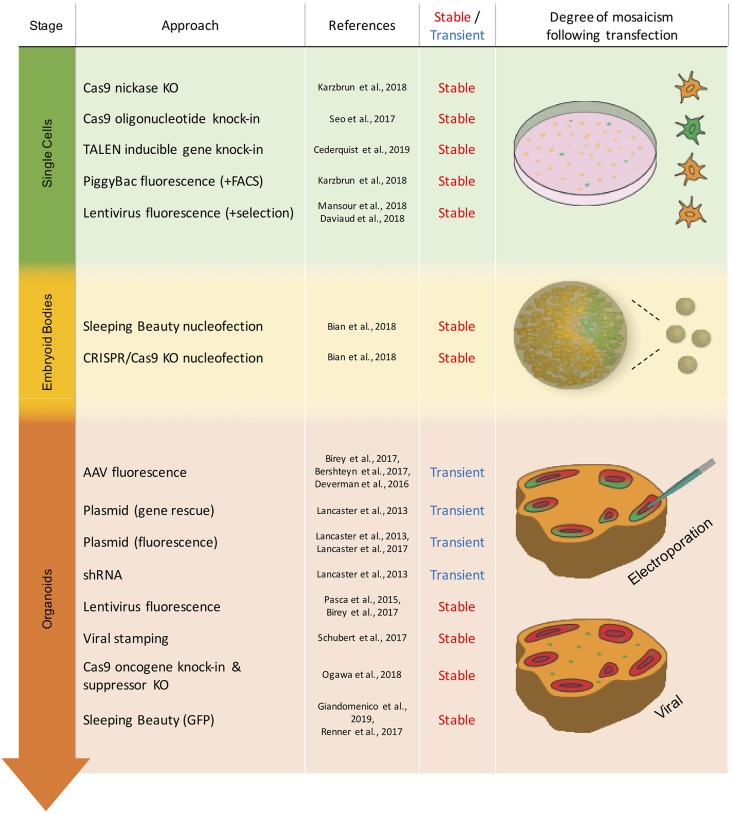
Overview of the transient and stable approaches for genetic modification in brain organoids. Techniques are listed by the stage of organoid development in which they have been used. Schematic representation of mosaicism and spatial distribution of genetically modified cells (green) following respective modification approaches.

**Figure 2:**
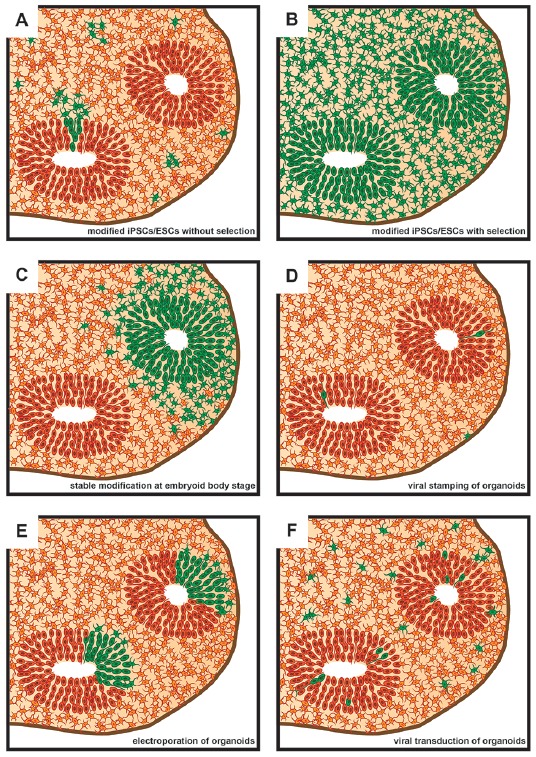
Schematic representation of sections of brain organoids as seen subsequent to various approaches of genetic modification. Green denotes genetically modified cells. **(A)** Brain organoid derived from genetically modified induced pluripotent stem cells (iPSCs)/embryonic stem cells (ESCs) that did not undergo subsequent positive selection. **(B)** Brain organoid derived from genetically modified iPSCs/ESCs that underwent subsequent positive selection using FACS or antibiotic resistance. The vast majority of cells within the brain organoid will contain the genetic modification. **(C)** Brain organoids derived from an embryoid body (EB) that underwent regional genetic modification through the use of an electroporation-based approach. Note that a ventricle-like structure will only show genetic modification if transfection efficiency and cell survival is sufficiently high in the targeted region of the EB. **(D)** Brain organoid following genetic modifications being targeted to single cells using viral stamping. Cells both at and away from, the brain organoid surface can be targeted. **(E)** Brain organoid following the electroporation of genetic cargo into ventricle-like structures. **(F)** Brain organoid following virus-based (e.g., lentivirus or adeno-associated virus, AAV) genetic modification approaches. The proportion of genetically modified cells can vary greatly depending on incubation times of brain organoids in medium containing viral vectors.

The second issue concerns the timepoint of the genetic modification relative to the stage of brain organoid development, which can range from the starting cell line over the embryoid body (EB) stage to mature brain organoids (Figure 1). The choice of this timepoint depends mainly on the purpose of the experiment and the proportion of cells to be affected by the genetic modification (Figure 2). Stable modifications are mostly performed at an early stage of brain organoid development, such as in the starting cell line, whereas transient modifications are typically performed at later stages, given the decline of genetic cargo levels due to cell proliferation (Figure 1).

Due to the large cellular heterogeneity of brain organoids, the third issue concerns the cell type(s) that is/are targeted within the organoid by the genetic modifications. Approaches can either target cells indiscriminately, regardless of their location and cell type or, contrariwise, target a subset of cells. This can be achieved either through direct visual identification of cells or alternatively by biologically restricting the genetic modification to specific cell types. Visual identification of target cells can be used for single-cell microinjections (Chow et al., 2016; Shull et al., 2019) or approaches such as viral stamping (to be discussed below; Schubert et al., 2018). Biological restriction, on the other hand, makes use of cell type-specific promotors to activate or suppress gene expression (Pasca et al., 2019; Birey et al., 2017). In the following, we will first discuss the various types of transient genetic modifications of brain organoids, and then the various types of stable genetic modifications.

## Transient Genetic Modifications of Brain Organoids

In transient genetic modifications of brain organoids, gene vectors are used that allow protein expression or the production of short interfering RNAs (siRNAs; Lancaster et al., 2013) for a limited amount of time. Transient expression vectors have therefore been almost exclusively administered into late-stage brain organoids rather than into the cells or EBs that precede them (Figure 1). For this type of genetic modification, one of the most important aspects to consider is the mode of plasmid delivery. The two most widely used methods in the case of brain organoids are viral delivery *via* adeno-associated viruses (AAVs) and non-viral delivery *via* electroporation.

### Viral Delivery—Adeno-Associated Viruses (AAVs)

AAVs are known to generally produce transient, rather than stable, expression of genetic cargo (e.g., genes or siRNAs). This is in contrast to the frequently used lentiviruses (see below), which typically produce stable transfections. However, it is important to note that AAVs can integrate genes into the genome of targeted cells at very low rates, particularly in dividing cells and at the AAV-safe loci (AAVS1; Deyle and Russell, 2009). AAVs have been successfully used in brain organoids to achieve fluorescence specifically in neurons (Deverman et al., 2016; Bershteyn et al., 2017; Birey et al., 2017). These experiments avoided targeting proliferative cell populations and primarily focused on neuronal migration within brain organoids. However, in organoid slice cultures, proliferative cell populations have been targeted using adenoviruses, which allowed for detectable fluorescence in these cells for 2–5 days (Bershteyn et al., 2017).

In most cases, AAVs are simply added to the cell culture medium, which results in scattered mosaic expression throughout the entire organoid or slice (Figure 2F). However, this mode of administration precludes a more focused targeting of individual regions within the brain organoid. Further drawbacks of AAVs include: (i) the need of special facility-requiring safety precautions; (ii) a greater amount of work in comparison to non-viral delivery due to the production and titration of virus particles; and (iii) a limit of 5 kilobases of cargo DNA (Grieger and Samulski, 2005).

### Non-viral Delivery—Electroporation

An important alternative to genetic cargo delivery *via* AAVs is electroporation. Analogous to *in utero* electroporations of embryonic mouse (Saito and Nakatsuji, 2001; LoTurco et al., 2009) and ferret (Kawasaki et al., 2012) neocortex, in which plasmid DNA is injected into the brain ventricles prior to the electroporation, ventricle-like structures in brain organoids can be identified and injected with plasmid DNA and subsequently electroporated (Lancaster et al., 2013; Li et al., 2017). Here, electrical pulses lead to pore formation in the cell’s plasma membrane to allow for plasmid uptake (Kar et al., 2018). However, particularly in older brain organoids (>2 months old), targeting of ventricles by injection becomes increasingly difficult due to a reduction in tissue translucency. This can partly be overcome by confining the manipulation to the outer regions of a brain organoid where ventricles are located, which can be blindly targeted by injection. However, this approach frequently results in increased mechanical damage of the brain organoid and decreased transfection efficiency (our own observations).

When the ventricles of brain organoids can be specifically targeted, electroporation has a much higher transfection efficiency than genetic cargo delivery *via* AAVs. Analogous to *in utero* electroporation, efficiencies of up to almost half of the cells lining the ventricular surface of the targeted region can be achieved (dal Maschio et al., 2012). Such high efficiencies allow for regionally confining the area of cell transfection within a given ventricular structure of the brain organoid (Figure 2E), depending on electrode placement. Thereby a comparison between transfected and untransfected areas within the same ventricular structure in the brain organoid can be made. A further benefit of electroporation over viral delivery is that cells are transfected at the same timepoint, whereas upon viral delivery transfection occurs over a temporal range. Yet, these benefits come at the risk of cellular toxicity due to the electrical pulses that are applied. However, if the voltages for electroporation are kept low (≤80 V), cell viability usually remains high (Lancaster et al., 2013). Importantly, reflecting the decline of genetic cargo levels over time prior to EB embedding, transient genetic modification using electroporation has been found to be limited to mature brain organoids (Lancaster et al., 2013; Li et al., 2017; Figure 1).

## Stable Genetic Modifications of Brain Organoids

Whilst transient genetic modification of brain organoids is suitable for many experimental approaches, it is insufficient for setups in which proliferative cells require modifications for longer periods of time. Not only can stable genetic modification approaches resolve this issue, but if applied to the brain organoid founder cells they can also greatly reduce mosaic expression patterns (Figures 2A,B). Stable genetic modification methods also heavily rely on viral delivery or electroporation (although the use of lipofection is also conceivable) in order to introduce genetic cargo into the cells. As above, genetic cargo is any nucleic acid from an exogenous source that is delivered into the cells to bring about a change in gene expression. However, unlike transient genetic modification, stable genetic modification is typically achieved by delivering a piece of machinery into the cells that subsequently will mediate the introduction of genetic modifications (from point mutations to integration of transgenes) into the host cell’s genome. A variety of tools that involve such a machinery have been developed to achieve genomic integration into the cells forming brain organoids, namely viruses with reverse transcriptase activity (lentiviruses), transposon systems, and CRISPR/Cas9.

### Viral Delivery—Lentiviruses

Retrovirus-based, in particular, lentivirus-based, transduction is an efficient, fast and easy-to-use method to achieve stable genetic modification of cells. Replication-deficient lentiviral vectors are one of the most widely used tools for this purpose (Janssens et al., 2019). These commonly are human immunodeficiency viruses pseudotyped with a vesicular stomatitis virus envelope, which allows infection of most cell types (Vigna and Naldini, 2000; Merten et al., 2016). In this system, the genetic cargo (referred to as transfer plasmid) and the viral components (structural proteins and the enzymes needed for the integration of the genetic cargo, collectively referred to as packaging plasmids) are contained on different plasmids. To generate functional viral particles, these plasmids are introduced into a packaging cell line, typical HEK293, and the thus generated viral particles are then collected from the cell culture supernatant. These viral particles contain only the genetic cargo but no genes for the packaging machinery, which implies that after infection of the target cells, the genetic cargo can be integrated into the host genome, but new viral particles cannot be produced by the infected cell (Vigna and Naldini, 2000).

Lentiviral vectors have been administered to brain organoids to induce fluorescence in target cells through the use of cell-type-specific promotors (Pasca et al., 2019; Birey et al., 2017). Due to relatively low transfection efficiencies, significant mosaicism results from the use of these vectors when they are simply added to the cell culture medium of brain organoids (Figure 2A). To overcome this, brain organoid founder cells can be virally targeted, with subsequent antibiotic selection or fluorescence-activated cell sorting in order to obtain a more homogeneous cell pool for brain organoid generation (Daviaud et al., 2018; Mansour et al., 2018; Janssens et al., 2019; Figure 2B).

Moreover, researchers will in the future not only be limited to the aforementioned approaches using lentiviral vectors, which infect without cell-type specificity, on bulk cell populations or tissue. Rather, selected single cells can be targeted using such viral vectors (Schubert et al., 2018; Figure 2D). This procedure makes use of a technique termed viral stamping, in which viral vectors are brought into direct physical contact with single target cells (Schubert et al., 2018). This approach is not limited to targeting cells at the surface of brain organoids, but can also target those that lie within them. In the latter case, using a so-called shielded approach, the viral particles, which are linked to magnetic nanoparticles, are only exposed to cell surfaces upon an electromagnetic pulse. Using viral stamping, individual cells within brain organoids have been successfully targeted at a depth of up to 150 μm from the organoid’s surface, with an efficiency of 10%–25% of the targeted cells (Schubert et al., 2018).

Regardless of approach, however, lentiviral vectors have several notable drawbacks. Although lentiviral vectors have a greater size capacity for genetic cargo than AAVs, only 10–12 kb of genetic cargo have been efficiently transduced, thereby placing limits on more complex gene constructs (Kumar et al., 2001; Counsell et al., 2017). A further disadvantage is that random genomic insertion of genes can lead to unwanted and unpredictable effects, ranging from cell apoptosis to uncontrolled proliferation through activation of proto-oncogenes.

### Non-viral Delivery

In contrast to viral delivery through lentiviruses, which introduces into the host cell both the genetic cargo and the machinery for its integration into the genome, non-viral delivery mediated by electroporation or lipofection may introduce only genetic cargo into the host cell. If so, non-viral delivery needs to include, or be combined with, introducing machinery into the host cell that mediates the integration of the genetic cargo into the genome. The two most often used such machinery are transposon-like systems and nuclease-based tools (nowadays mainly CRISPR/Cas9).

### Transposon-Like Systems

Transposon-like systems used to obtain stable genetic modification are based on mobile DNA elements. These elements can move (“transpose”) their position within the genome using a “cut and paste” mechanism (McClintock, 1950; Grabundzija et al., 2010). This is made possible by the presence of a transposase that is able to recognize inverted terminal repeats (ITRs) flanking the transposon, which is then excised and inserted at a different site. In current transposon-like systems, the DNA cargo of interest is tagged with flanking ITRs, and the appropriate transposase is provided in trans, leading to the integration of the DNA cargo into the target genome (Ni et al., 2008). Two of the most notable transposon-like systems that have been developed are Sleeping Beauty (Ivics et al., 1997) and PiggyBac (Ding et al., 2005; Wilson et al., 2007).

With regard to brain organoids, these transposon-like systems have been used not only to achieve GFP reporter expression (Lancaster et al., 2017; Renner et al., 2017; Karzbrun et al., 2018; Giandomenico et al., 2019), but also to introduce oncogenes (Bian et al., 2018). With generally higher genomic insertion efficiencies than retroviral vectors (Ding et al., 2005; Chen et al., 2015), transposon-like systems can be delivered, typically *via* electroporation, not only into brain organoid founder cells, often followed by selection of expressing cells, but also into EBs (Bian et al., 2018) and even mature organoids. The aim of this approach is to achieve expression in targeted cell populations and their progeny within the brain organoid (Figure 2C).

Despite several advantages, including the potentially large size of the transposable elements of up to 14 kb, some notable drawbacks of transposon-like systems exist (Ding et al., 2005). Thus, uncertainty as to the level of gene expression when using these systems can be a major disadvantage. Specifically, transgenes may have integrated a variable number of times into the genome of a given cell type, leading to significant differences in expression from cell to cell. Furthermore, the genomic integration of the cargo DNA can interfere with the expression of endogenous genes, leading to unwanted side effects.

### CRISPR/Cas9

An alternative to achieve stable genetic modification is to make use of nuclease-based tools. A variety of nuclease-based tools have been developed to induce targeted mutations and gene insertions, ranging from Transcription Activator-Like Effector Nucleases (TALENs; Boch et al., 2009) and Zinc Finger Nucleases (ZFNs; Kim et al., 1996) to the now very popular Clustered Regularly Interspaced Short Palindromic Repeats/Cas9 (CRISPR/Cas9; Jinek et al., 2012; Cong et al., 2013; Mali et al., 2013) system. Although there is still some use of TALENs (Cederquist et al., 2019) and ZFNs in the context of stable genetic modifications of brain organoids, these nuclease-based tools have now largely been replaced by the CRISPR/Cas9 system due to its relative ease of use and high efficiency. This system uses a guide RNA to target the nuclease Cas9 to a specific genomic locus to then cause a double-strand break, thereby activating endogenous DNA repair processes. These can be non-homologous end joining (NHEJ) or homologous-directed repair (HDR). NHEJ has a high propensity for generating small deletions or insertions during repair, thus enabling the generation of knockouts or knockdowns of targeted genes. HDR, on the other hand, is useful for introducing changes (ranging from point mutations to transgene insertions) in the presence of a DNA template to instruct the repair DNA synthesis (Doudna and Charpentier, 2014; Harrison et al., 2014; Hsu et al., 2014). It is, however, important to note that NHEJ has also been shown to play an important role in the insertion of transgenes particularly in postmitotic cells which show significantly reduced HDR (Suzuki et al., 2016; Suzuki and Belmonte, 2018). CRISPR/Cas9-mediated genetic modifications are usually conducted on brain organoid founder cells, that is, ESCs and iPSCs (Bershteyn et al., 2017; Iefremova et al., 2017; Li et al., 2017; Matsui et al., 2017; Fiddes et al., 2018; Karzbrun et al., 2018). Cells containing the desired genomic change are then selected and can be used to grow brain organoids. This approach has the advantage that all cells in the organoid will contain the previously introduced genetic modification (Figure 2B).

However, the use of CRISPR/Cas9-mediated genetic modifications is not limited to brain organoid founder cells. Plasmid vectors containing Cas9 along with one or more guide RNAs, or *in vitro* formed complexes of recombinant Cas9 protein and guide RNAs (Kalebic et al., 2016), can be electroporated directly into EBs or early organoids to result in loss of function mutations (Bian et al., 2018). This has been explored in organoid models of central nervous system (CNS) tumors in which tumor suppressor genes were targeted to provoke neoplasia (Bian et al., 2018). A further example is the combined use of a CRISPR/Cas9-directed tumor suppressor (TP53) knock-out combined with a CRISPR/Cas9-directed oncogene (KRAS) knock-in in 4 months-old human brain organoids (Ogawa et al., 2018). By generating cells with a glioblastoma-like proliferative potential, only a few cells needed to successfully undergo stable genetic modification.

In certain cases, especially for knock-outs of genes that exert essential functions at certain stages of brain organoid development, it would be beneficial to have temporal control of CRISPR/Cas9 activity. To this end, one may consider using CRISPR/Cas9 as an inducible system. For example, Tet- or Lac-based systems could be combined with Cas9 and guide RNA (Sun et al., 2019). Once this system is introduced into the ESC or iPSC genome, it would allow for a time-specific knockout of the gene of interest. Transcription of Cas9 would be induced by the addition of doxycycline (Tet) or isopropyl ß-D-1-thiogalactopyranoside (Lac) to the culture medium.

Of note, Cas9 activity in cells over long periods of time increases the risk of off-target effects, thereby having unforeseen effects on cell survival and phenotype. To reduce such effects, paired Cas9 nickases can be used (Ran et al., 2013). Cas9 nickases can only produce single-strand cuts rather than the double-strand cuts of wild-type Cas9. This means that two nickases need to target sequences in very close proximity to one another to result in a successful genetic modification event. This significantly increases target specificity, albeit at the cost of genetic modification efficiency.

One prime application of the CRISPR/Cas9 system in brain organoids is its use in modeling neurodevelopmental and neurodegenerative diseases. Here, either patient mutations can be introduced into control iPS cell lines, or patient iPS cells can be “repaired” to generate isogenic controls (Iefremova et al., 2017; Matsui et al., 2017; Fiddes et al., 2018). This was for example successfully applied in the modeling of retinoblastoma (Matsui et al., 2017) and Miller-Dieker Syndrome (Bershteyn et al., 2017; Iefremova et al., 2017).

## Outlook and Future Applications

In summary, different modes and methods of genetic modifications have been successfully applied at various time points of brain organoid development. The spectrum of potential applications has so far ranged from the simple expression of fluorescent marker proteins (Pasca et al., 2019; Renner et al., 2017) to the study of gene function (Lancaster et al., 2013) to the modeling of disease conditions (Bershteyn et al., 2017; Iefremova et al., 2017; Matsui et al., 2017). Yet, significant untapped potential still remains.

In the case of transient genetic modification, the electroporation of mature organoids—although already successfully applied in the first report of cerebral organoids (Lancaster et al., 2013)—is not frequently employed. Many labs successfully utilize *in utero* electroporation of mice (Saito and Nakatsuji, 2001; LoTurco et al., 2009) and less frequently rat (Szczurkowska et al., 2016) and ferret (Kawasaki et al., 2013) developing neocortex to study gene function. Brain organoids could provide a potential replacement here, especially if their capacity to model 3D neural tissue is further improved. Moreover, the use of human or chimpanzee brain organoids (Mora-Bermúdez et al., 2016; Otani et al., 2016; Heide et al., 2018; Kanton et al., 2019; Pollen et al., 2019) for electroporations represents a huge opportunity as these organoids provide an environment that is comparable to the early stages of fetal human neocortex development in terms of gene expression and cell-type composition (Camp et al., 2015; Velasco et al., 2019). This could prove to be particularly beneficial for the study of human-specific genes that are expressed during cortical development either in progenitors or newborn neurons (Florio et al., 2018; Suzuki et al., 2018). Electroporation of chimpanzee brain organoids would be a relatively quick and powerful test to examine the function of these genes and would be one of the very few possible ways to study them in our closest living relative of the Hominidae family.

In the case of stable genetic modification, CRISPR/Cas9 is, and most likely will be, the method of choice to introduce such modifications in brain organoids. It is easy to use, does not require special safety precautions and is very efficient in comparison to other targeted genetic modification techniques. At present, a major application of CRISPR/Cas9 in brain organoids is the generation of disease models and is primarily focused on monogenic neurodevelopmental diseases as well as oncogenic mutations. A major future challenge will be to study more complex disease states that are oligo- or polygenic in nature.

Nonetheless, viral and transposon systems will still play a significant role in future brain organoid studies. This is due to the versatility of these tools and their greater genetic modification efficiencies in comparison to CRISPR/Cas9, particularly in EBs and mature brain organoids. Notably, the use of viruses in combination with viral stamping may prove useful in tracing axonal connections within single brain organoids as well as organoid assembloids (Pasca, 2019; Schwarz and Remy, 2019). Furthermore, advanced techniques to control the behavior of individual neurons, such as optogenetics, should also open up new avenues for brain organoid studies (Frank et al., 2019). In this context, the demonstration of oscillating electrical waves within cortical organoids (Trujillo et al., 2019) is of major relevance.

A major focus of future applications of stable genetic modifications of brain organoids will likely be cell lineage tracing. A multitude of methods have been devised in various systems that employ CRISPR/Cas9, viral vectors or transposons in order to track the developmental origin and fate of individual cells (Figueres-Oñate et al., 2016; Kebschull and Zador, 2018; McKenna and Gagnon, 2019). These methods make use of a differential expression of fluorophore combinations or other cell barcoding techniques. As to brain organoids, these approaches of cell lineage tracing could constitute a vital experimental avenue to confirm and complement *in silico* lineage analyses derived from single-cell sequencing (Camp and Treutlein, 2017; Camp et al., 2018). A significant advance in this context would be the transcriptomic analysis of defined cell subpopulations with an identified location within brain organoids, labeled *via* lineage-specific expression of fluorescent proteins.

Finally, combinations of stable and transient genetic modifications will likely hold great promise for studying various aspects of brain organoid development and performance in the future. For example, such combination may prove advantageous for rescue experiments of a given phenotype. On a general note, combinations of stable and transient genetic modifications will allow for the modeling and dissection of increasingly complex disease states as well as developmental and evolutionary processes in brain organoids.

## Author Contributions

All authors wrote and edited the manuscript.

## Conflict of Interest

The authors declare that the research was conducted in the absence of any commercial or financial relationships that could be construed as a potential conflict of interest.
